# The Incredible Years Parents and Babies Program: A Pilot Randomized Controlled Trial

**DOI:** 10.1371/journal.pone.0167592

**Published:** 2016-12-14

**Authors:** Maiken Pontoppidan, Sihu K. Klest, Tróndur Møller Sandoy

**Affiliations:** 1 SFI–The Danish National Centre for Social Research, Department for Child and Family, Copenhagen, Denmark; 2 Department of Public Health, University of Copenhagen, Copenhagen, Denmark; 3 Health Sciences Faculty, University of Tromsø, Arctic University of Norway, Tromsø, Norway; TNO, NETHERLANDS

## Abstract

**Introduction:**

Infancy is an important period of life; adverse experiences during this stage can have both immediate and lifelong impacts on the child’s mental health and well-being. This study evaluates the effects of offering the Incredible Years Parents and Babies (IYPB) program as a universal intervention.

**Method:**

We conducted a pragmatic, two-arm, parallel pilot randomized controlled trial; 112 families with newborns were randomized to the IYPB program (76) or usual care (36) with a 2:1 allocation ratio. The primary outcome was parenting confidence at 20 weeks(Karitane Parenting Confidence Scale and Parental Stress Scale). Secondary outcomes include measures of parent health, parent-child relationship, infant development, parent-child activities, and network. Interviewers and data analysts were blind to allocation status. Multiple linear-regression analyses were used for evaluating the effects of the intervention.

**Results:**

There were no intervention effects on the primary outcomes. Only one effect was detected for secondary outcomes, intervention mothers reported a significantly smaller network than control mothers (β = -0.15 [-1.85,-0.28]). When examining the lowest-functioning mothers in moderator analyses, we found that intervention mothers reported significantly higher parent stress (β = 5.33 [0.27,10.38]), lower parenting confidence (β = -2.37 [-4.45,-0.29]), and worse mental health than control mothers (β = -18.62 [-32.40,-4.84]). In contrast, the highest functioning intervention mothers reported significantly lower parent stress post-intervention (β = -6.11 [-11.07,-1.14]).

**Conclusion:**

Overall, we found no effects of the IYPB as a universal intervention for parents with infants. The intervention was developed to be used with groups of low functioning families and may need to be adapted to be effective with universal parent groups. The differential outcomes for the lowest and highest functioning families suggest that future research should evaluate the effects of delivering the IYPB intervention to groups of parents who have similar experiences with parenting and mental health.

**Trial registration:**

ClinicalTrials.gov NCT01931917

## Introduction

Increasing numbers of studies with small children suggest that the youngest are at the highest risk of serious developmental harm. During infancy, while children are at their most malleable, they are also exposed to more potentially damaging experiences than older children. For example, the highest rates of child neglect and violent abuse occur when children are under five years old [1,2], with the most serious injuries and death caused by parental violence against children when infants are under one year old [3].

Infants are also more sensitive to disruptions in parental care than older children. Infants with mothers who suffer from severe stress or depression can show physiological, biochemical, and behavioral dysregulations shortly after birth, and have been found to be at increased risk of behavioral and mental problems [4]. These infants often show avoidance of caretakers and display high levels of distress and negative emotion [4]. For many of these children, these effects are the beginning of a trajectory of negative developmental and mental health outcomes throughout childhood and adulthood [5,6].

Early parent-child interactions have been shown as the key predictive factor for many early and late developmental outcomes [7]. Supporting parents in developing and applying positive parenting skills can prevent future problems and encourage healthy child development [8–16]. This study evaluates a program of parental support based on these child development goals.

The new program, specifically designed for parents with infants, has been added to the Incredible Years Series (IY) of treatment and prevention programs for children and families. Extensive Scandinavian and international research, from both the program developer and independent research groups, has demonstrated the effectiveness of the IY programs for older children [9,17–34].

A recent meta-analysis of IY parenting programs for three- to nine-year-old children shows that effect sizes for parent-reported outcomes in treatment studies (families who sought help) were higher (*d* = 0.50) than for indicated prevention studies (families with children with minimal symptoms) (*d* = 0.20) and selective prevention studies (families with high risk) (*d* = 0.13) [28]. A shortened version of the universal Basic IY parent program for children aged 3–8 was evaluated in an RCT, which showed reductions in harsh parenting, parent stress, and parent depression, and increases in positive parenting, and parent sense of competence; improved parenting was maintained four years after the program ended [16,35]. The IY Toddler BASIC program for children aged 1–3 has been evaluated in three trials with positive results [22,36,37], however, we still know less about the effects of the IY programs for children under three than we do for older children [29].

In 2010, the developer of the IY program extended the series to include the Incredible Years Parents and Babies program (IYPB) for families with infants from birth to one year. IYPB has not been evaluated for effectiveness in a randomized controlled trial (RCT), either in Scandinavia or internationally. However, a pre-post evaluation of IYPB in Wales found that both parenting competence and mental health significantly improved over time for those who participated in the program[38]. A second evaluation conducted in Wales with a control group, also found that mothers in both treatment and control groups significantly improved over time. A significant positive effect on observed mother sensitivity was identified, while no differences were found between the two groups on child development, parenting confidence, or parental mental well-being [39].

During the eight-week course of treatment in IYPB, mothers and fathers attend weekly group sessions with their infants. Group leaders (therapists) trained in IYPB conduct the sessions with approximately eight families. During the sessions, parents learn to observe, read, and respond in a sensitive manner to their babies’ cues and signals. They also learn how to understand babies as intelligent learners, how to provide physical and tactile stimulation for babies, how to take care of their own needs as parents, how to understand babies’ developmental processes and needs, and the importance of good care for infant brain development.

In Scandinavia, no programs with strong evidence of effectiveness are available for the treatment and prevention of developmental problems in infants (e.g., insecure attachment). The Ungsinn database (ungsinn.no), which is used for cataloging and rating programs available for children in Norway, lists only one parenting program for infants, and at present not enough evidence exists to support its effectiveness. In Denmark, the Circle of Security program is currently being evaluated in an RCT. Even fewer interventions, either in Scandinavia or internationally, are aimed at a universal sample of parents with infants [40–47]. Three of these programs are delivered postnatally in a group format: (1) Toddlers Without Tears (Australia), with three sessions from age eight months [45,48]; (2) Face to Face (Australia), with five sessions from three months [49]; and (3) an American trial of parent training, with eight sessions from age eight months [50]. None of the studies, however, finds any effects on child development or the parent-child relationship. When compared to these three interventions IYPB starts at an earlier age and offers significantly more sessions than Toddlers Without Tears and Face to Face. In this pilot RCT, we evaluate a more intensive intervention, offered as a universal intervention aimed at a community sample of parents with newborns.

The aim of this pilot trial was to evaluate the effects of the program offered as a universal intervention in Denmark on the parent-infant relationship, parent and infant well-being, infant development, and to establish parameters for a future full-scale trial. The secondary aims were to provide information on the usability of parent and infant measures, to test recruitment procedures and determine rates of recruitment and parent consent, to investigate the implementation and parents’ acceptance of IYPB in a universal setting, and to provide information on the cost of offering IYPB as a universal preventive program.

## Methods

### Study Design

The trial was a pragmatic, two-armed, parallel pilot RCT. Institutional review board approval was obtained from SFI–the Danish National Center for Social Research. Parents provided informed consent before participation. The trial was carried out according to CONSORT guidelines [51,52] and registered at ClinicalTrials.gov (reference number NCT01931917).

### Participants and Recruitment

The eligible participants were mothers with infants living in the Ikast-Brande or Herning local authority area in Denmark. Where fathers were present, they were also invited to participate. All mothers in Denmark are entitled to 46 weeks of maternity leave following the birth of a child; the last 32 weeks can be split with the father. Virtually all families receive five or six free home visits from health visitors and three child-health visits to the general practitioner within the child’s first year [53,54]. Health visitors recruited families between August 2013 and August 2015. After the local contact person received initial consent from the family, the interviewer arranged a home visit, during which written consent was obtained from the mother (and the father, if applicable).

### Inclusion Criteria

The trial included mothers who are able to read and write Danish and had infants aged 0–4 months. Ikast-Brande recruited only first-time mothers, whereas Herning included all mothers. Although families could have been excluded in the case of severe physical or mental disability in the parent or the child, or if the child was placed in out-of-home care, no families were excluded from participation.

### IYPB Intervention

The IYPB program was developed to promote babies’ physical, emotional, and language development. The intervention aims at promoting a warm, nurturing parent-child relationship, and enhancing parent competencies [55]. Groups consisted of 6–8 parents and were led by two trained group leaders. Mothers brought their babies to the sessions, and partners were strongly encouraged to participate. The purpose of the group format is to stimulate shared learning and peer support networks. Parents practiced new skills with babies within the group, and were encouraged to try out new solutions at home as part of their weekly assignments [55].

The program consisted of eight two-hour sessions. To support the training and foster discussion in the group, during each session, group leaders showed video vignettes of real-life situations with parents and babies. The original American video vignettes were used with Danish subtitles. Group leaders used the IY baby brain poster to explain the importance of infant brain development and demonstrate how parents strengthen neural connections and can support their child’s healthy brain development. Parents also received a Danish translation of the book *The Incredible Babies* [56], which describes how to promote positive child development and includes a journal section for parents to use. The six parts covered during the course are titled: (1) "Getting to Know Your Baby"; (2) "Babies as Intelligent Learners"; (3) "Providing Physical, Tactile and Visual Stimulation"; (4) "Parents Learning to Read Babies’ Minds"; (5) "Gaining Support", and (6) "Babies’ Emerging Sense of Self" [55]. The group leaders followed a manual to ensure that the intervention was performed with fidelity.

Two group leaders were certified IYPB group leaders and two were in the process of gaining IYPB certification. The remaining group leaders were all experienced IY group leaders who were certified in the IY BASIC Parent Group and who attended three days of training sessions in IYPB. Group leaders attended supervision twice a year with an IYPB mentor.

### Control

The families randomized to the control group received usual care (UC), consisting of four or five home visits from health visitors, open consultation hours at a local well-child clinic, voluntary participation in a social group of six local mothers, and extra support if needed (e.g., extra home visits, family therapy, or video-feedback intervention). Intervention families received IYPB in addition to UC.

### Measures

Outcomes were collected through home visits at baseline (T1) and post-intervention (T2). The mean number of days between T1 and T2 was 146 (SD 28), with a range of 78–207 days. Given the difference in the number of weeks that the IYPB program ran, the range was large. For some groups half of the IYPB sessions were offered before the summer or the Christmas break, with the other half offered afterwards. Other groups finished within a continuous 12-week period. T2 data was collected between one and three weeks after the IYPB program. Baseline measures and the timing of measures are described in the trial protocol [57]. While both mothers and fathers could complete the questionnaire, only a few fathers did so (50 at T1, 14 at T2). Families received a 200 DKK (≈$30) gift card at each visit.

The primary outcomes were the 15-item Karitane Parenting Confidence Scale [58,59] and the 18-item Parenting Stress Scale [60]. Secondary parental outcomes included the 10-item Major Depression Inventory (MDI10) [61], the five item WHO-5 Well-Being Index [62,63], the 10-item Rosenberg Self-esteem Scale [64], and single items on parent health, and parent life satisfaction, support, and network. Secondary child outcomes included the 26-item Ages and Stages Questionnaire: Social-Emotional-2 experimental version (ASQ:SE-2e) [65,66] and single items on child health, temperament, height, and weight. Secondary relationship outcomes included the 10-item Mother and Baby Interaction Scale (MABISC) [67,68], three single items measuring parent and child interaction through singing and reading, and a 15-minute video of the mother and baby. The videos have not yet been coded; therefore, the results for this outcome will be presented in a separate paper. In addition, we collected demographic characteristics such as parent age, education, occupation, primary language spoken in the home, number of children, household budget, substance abuse, birth weight, gestational age, and child health, and whether parents cohabit or the mother lives alone, and whether housing is rented or owned. All trial outcomes are described in greater detail in the study protocol [57].

### Randomization and Blinding

An independent researcher computed a random allocation list, stratified by the local authority, with a block size of three. A 2:1 (IYPB:UC) allocation ratio was used as a practical consideration as the providing health centers needed to have enough families allocated to the IY intervention to be able to start a group before the infants grew to old. Following completion of the baseline assessment, the interviewer informed a designated research administrator that the interview had concluded. The research administrator then randomized the families by adding their names to the randomization list in the order that the interviewer had provided. The health visitor informed each family which arm of the study they were allocated to. In cases where consent to treatment was withdrawn but the participant agreed to remain in the research study, the participant was followed to completion. Given the nature of the trial, participants and group leaders could not be blinded to condition. Interviewers, coders, and data analysts were blind to group allocation status.

### Parent and Group Leader Satisfaction

Parents completed questionnaires after each session and a final evaluation after the last session. For Ikast-Brande municipality, final evaluation data from 82 parents who participated in the IYPB between January 2014 and July 2015 was analyzed [69]. Furthermore, qualitative interviews with group leaders and focus groups with parents were held in Ikast-Brande [70].

### Statistical Analyses

We aimed to recruit 128 mothers based on power calculations for a clinical sample; however, before the trial began, local political authorities mandated that all families be offered a place in the program. We did not have further resources to collect additional data for a larger sample or the required information to calculate power for a universal population, we therefore changed the focus to a pilot trial for a universal sample.

Categorical data is presented as numbers and percentages, and continuous data as means and standard deviations. For demographic data, we applied independent t-tests for testing differences for continuous variables, while Chi-square tests were applied for categorical variables. The trial included two sets of twins, both in the IYPB group. To account for the lack of independence between twins, we selected the first twin and ignored the other for parent outcomes but kept both twins for child outcomes. We performed intention to treat analyses for primary and secondary outcomes with multiple regression analysis, including controls for site and baseline score. No further covariates were included, as variables such as the mother’s age, education, and parity had no effect on the results.

Of the 112 mothers assessed at T1, eight were lost due to attrition at T2. Depending on the mechanism by which data went missing, attrition could lead to bias in the estimates. To deal with attrition, we first tested the assumption of data being missing completely at random (MCAR) by creating an indicator variable for observations missing at T2 and fitting it with a logit model. All baseline measures of the outcome variables and other covariates, along with the treatment dummy, were included as predictors. Although we found no significant predictors at the 5% level, five out of 18 predictors were significant at the 10% level, indicating that the data was missing at random (MAR). Although the assumption of MAR data is not testable, imputation is still shown to produce less biased results than listwise deletion [71]. Furthermore, should the data really be MCAR instead of MAR, listwise deletion would lead to unbiased, albeit possibly inefficient, estimates. As a result, we generated 200 datasets with multiple imputation [72] using Stata’s multiple imputation (mi) impute procedure on chained equations.

Although not all variables were normally distributed, we applied the ordinary least squares (OLS) regression, as the OLS is consistent even without normality. A two-tailed test α = 0.05 was implemented for all analyses. We calculated effect sizes by dividing the adjusted mean difference between the trial arms by the pooled standard deviation. We used robust standard errors to account for group effects. We applied paired sample t-tests for testing total group change over time. As described in the protocol, we examined differential effects by comparing IYPB to UC in three subsamples: (1) parents with baseline scores in the clinical range of the measures, (2) parents scoring within the lowest 25% of the distribution at baseline, and (3) parents scoring within the lowest 50% of the distribution at baseline. For subsamples (2) and (3) we performed a moderator analysis, including an interaction term between a dummy for being in the 25% or 50% lowest/highest scoring group and a dummy for intervention allocation. Analyses were performed with Stata version 14.

## Results

**Fig 1:**
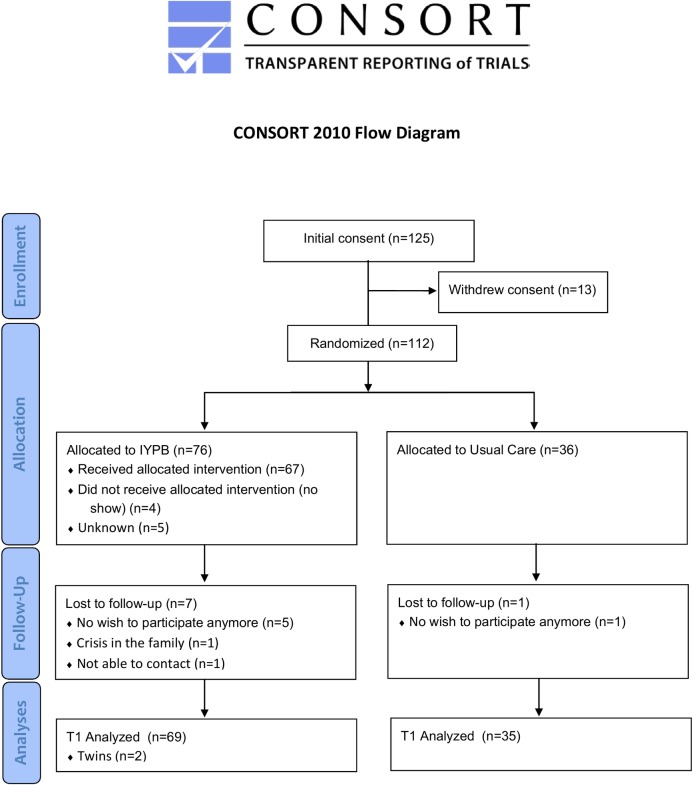
Trial flowchart.

Fig 1 presents the flow diagram of participants in the trial. Of the 125 families who had given their initial consent, 13 withdrew consent when the interviewer contacted them to schedule a home visit. Of the 112 families that were randomized, 76 were allocated to IYPB and 36 to UC. Eight families dropped out before T2 assessment. A larger dropout was observed in the intervention group (seven) than in the control group (one). Four of the seven mothers who dropped out of the study intervention group, did not start or dropped out of the IYPB group. Some statistically significant differences were found between the families who dropped out and those who did not; dropout mothers were older (5.76 years older on average, *p* = 0.0009), had more children (0.80 more children on average, *p* = 0.002), reported having attended more open house sessions with health visitors, and had a larger network at baseline for help with practical issues in the home.

Table 1 presents descriptive statistics for the two study conditions. We found no significant differences in demographic characteristics between IYPB and UC.

**Table 1:** Demographic characteristics at pre-intervention assessment for trial families who participated in the IYPB program and those who received usual care (UC).

		IYPB		UC	
		Mean	*SD*	Mean	*SD*
MOTHER	Age (years)	29.39	4.88	29.28	4.89
	Number of children	1.4	0.71	1.4	0.77
		***N***	**%**	***N***	**%**
	Mother living alone	7	9	3	8
*Education*	Low education	8	11	3	8
	Medium education	26	35	17	47
	High education	40	54	16	44
*Smoking*	Non-smoker	64	86	32	89
	Daily smoker	10	14	4	11
*Alcohol consumption*	>1 a week	1	1	1	3
	1 a week–1 a month	21	28	6	17
	Never or <1 a month	52	70	29	81
*Employment*	Working	54	75	26	72
	Student	10	14	3	8
	Unemployed	8	11	7	19
CHILD		**Mean**	***SD***	**Mean**	***SD***
	Age (months)	1.59	0.88	1.45	0.89
	Birthweight (grams)	3453	644	3549	499
		***N***	**%**	***N***	**%**
*Gender*	Boy	41	53	19	54
	Girl	33	45	17	47
*Gestational age*	28–31 weeks	1	1	0	0
	32–36 weeks	3	4	1	3
	37–42 weeks	64	86	29	81
	>42 weeks	6	8	6	17
FAMILY		***N***	**%**	***N***	**%**
	Danish first language	70	95	36	100
*Home-ownership*	Own home	45	61	21	58
	Rent home	29	39	15	42

IYPB: Incredible Years Parents and Babies; UC: Usual Care; *SD*: Standard Deviation

The mothers who participated in the trial appear to be relatively representative of the general population in relation to most demographic characteristics, with the exception of education level. When comparing the education level of the mothers in the trial with data from Statistics Denmark for all mothers in this age group (20–39), both in Denmark as a whole and for the two specific local authorities, Table 2 shows that the participating mothers generally had higher levels of education than the overall population.

**Table 2:** Distribution of education level (%) for Denmark, the Herning and Ikast-Brande communities as a whole where the study took place, and the trial sample.

Education	Denmark total %	Herning and Ikast-Brande %	Trial sample %
Primary school	16.05	17.45	10.00
High school/secondary	20.41	16.75	12.73
Vocational education/secondary	24.96	32.62	26.36
Short tertiary	4.49	7.44	9.09
College/bachelor/tertiary	23.45	21.73	32.73
University/long tertiary	10.24	3.95	8.18
Ph.D.	0.41	0.06	0.91

That the families who elect to participate in research studies often have more education than the population mean is a well-known tendency, one that has implications for the generalizability of trial results. Although the education level was higher than that of the general population, the mental health of the mothers appears to be representative of the general population. The total mean of mental health, as measured by WHO5 at T1, is 62.6 at baseline, a number lower than the mean of 67 for Danish women in general [73], but rose to 69.9 at T2. The level of clinical depression in the recruited mothers is 6% at T1, in line with the reported 7–8% for the Danish population when the child is five weeks old [74].

### Recruitment

Health visitors had different experiences with the recruitment process. Some found it challenging to ask families to participate in the trial, while others felt confident. During the trial, we asked the more confident recruiters at a meeting to share their strategies and experiences with the others. One of the two sites adopted a more insistent recruitment strategy, recruiting 62 mothers into seven IYPB groups (8.9 mothers per group). In contrast, the less insistent site recruited 50 mothers to 18 groups (2.8 mothers per group). Dropout rates were 10% for the insistent site and 4% for the less insistent site. The two-minute YouTube recruitment video was viewed 88 times, but we have no information on who viewed it or whether it was influential in the decision to participate in the study.

### Participation

While both sites registered parent participation, only one site provided full information on the number of completed sessions. Ikast-Brande recruited 50 families, 33 of which were allocated to intervention. Thirty mothers participated in the IYPB group, as three did not show up. On average, the participating mothers completed 6.3 sessions. Fathers participated less often than mothers;—a total of 17 fathers participated, and they completed four sessions on average. We found no difference between IYPB and UC in terms of either the number of extra visits families received from health visitors or the proportion of families who received extra visits in Ikast-Brande. IYPB families received on average 1.48 extra visits; UC families received on average 1.82 extra visits. A total of 33% of IYPB families and 29% of UC families received no extra visits. For Ikast-Brande, IYPB did not reduce the number of extra home visits families received. The protocol describes Complier Average Causal Effects (CACE) analyses for two levels of participation: (1) mothers who participated in at least three of the eight sessions, and (2) mothers who participated in at least six of the eight sessions. However, as we were able to obtain detailed participation data from only one site, we could not perform these analyses.

### Effect Evaluation

Table 3 shows the means for the full trial sample at T1 and T2. All outcome measures based on rating scales significantly improve from T1 to T2, except for self-esteem (RSS). Most single items (loneliness, confidants, parent health, child health, overall life satisfaction) do not change over time. One of the single items(parent report of child temperament)improves significantly over time, whereas mother reports of both family budget and network for help with practical issues decline significantly from T1 to T2. Because some of the measures were not strictly normally distributed we performed a sensitivity analysis by applying a non-parametric test (Wilcoxon signed rank test) to the non-imputed data. With the exception of self-esteem, which significantly improves over time (p = 0.02), results are consistent with the t-test.

**Table 3:** Means and standard deviations at pre-intervention (T1) and post-intervention (T2) for the full trial sample. Change for the full sample from T1 to T2 assessed with t-test p-value and Cohen’s d.

	**T1 All**		**T2 All**				
	Mean	SD	Mean	SD	Δ	p	*d*
KPCS	41.09	3.21	42.55	2.20	1.46	**<0.000**	0.54
MDI ¤	8.33	5.89	6.22	5.33	-2.11	**0.006**	-0.39
WHO5	62.62	16.73	69.86	15.70	7.24	**0.001**	0.47
RSS	24.86	4.52	25.69	4.59	0.83	0.180	0.17
ASQ:SE2e ¤	48.17	23.47	25.54	16.13	-22.63	**<0.000**	-1.15
*Single items*							
Loneliness	7.06	2.49	7.19	2.44	0.13	0.706	0.01
Network	8.77	2.02	8.03	2.77	-2.74	**0.020**	-0.28
Confidants	9.43	2.07	9.67	0.94	0.24	0.272	0.16
Overall health self-report	8.61	1.33	8.75	1.27	0.14	0.414	0.11
Life satisfaction	9.13	1.10	9.07	0.99	-0.06	0.691	-0.07
Budget	7.71	1.71	7.05	2.21	-0.66	**0.014**	-0.39
Child temperament	8.77	1.51	9.26	1.19	0.49	**0.007**	0.40
Child overall health	9.44	0.90	9.44	1.21	0.00	0.981	-0.01

**Bold** items are significant at *p* < 0.05; T1: Time 1 (pre-intervention); T2: Time 2 (post-intervention); SD: Standard Deviation; Δ: T1–T2 change; p: p value of paired t-test; d: Cohen’s d

¤: low score is favorable

KPCS: Karitane Parenting Confidence Scale; PSS: Parenting Stress Scale; MDI: Major Depression Inventory; WHO5: Well-Being Index; RSS: Rosenberg Self-Esteem Scale; MABISC: Mother and Baby Interaction Scale; ASQ:SE-2e: Ages and Stages Questionnaire: Social-Emotional-2 experimental version

Table 4 shows means and regression output comparing IYPB mothers and UC mothers. The baseline scores for intervention and control mothers do not differ for any of the outcomes apart from one item (child health), for which intervention mothers report significantly better child health than control mothers. However, this difference is not clinically significant, as both groups report a mean above nine on a scale 0–10. A comparison of IYPB and UC mothers at program completion shows no significant difference in any of the outcomes except one: “Mother report of a network that can help with practical issues” is significantly lower for mothers in the IYPB group than in the UC group at T2 (β = -1.07 [-1.85,-0.28], *d* = -0.18).

**Table 4:** Comparison of parent and child outcomes for families who received IYPB and usual care (UC) pre-intervention (T1) and post-intervention (T2) with regression coefficients, 95% confidence intervals, and effect sizes for multiple linear regressions on imputed data controlling for site and baseline score.

	IYPB		UC				
	T1	T2	T1	T2			
	Mean	Mean	Mean	Mean	β	CI	*D*
KPCS	41.30	42.60	40.67	42.45	-0.08	[-0.81,0.65]	-0.02
PSS ¤	-	30.76	-	30.05	0.79	[-1.85,3.42]	0.05
MDI ¤	7.65	6.05	9.72	6.56	0.36	[-1.36,2.09]	0.03
WHO5	64.16	69.95	59.44	69.68	-1.48	[-6.55,3.60]	-0.04
RSS	25.35	25.93	23.86	25.19	-0.17	[-1.63,1.29]	-0.02
MABISC ¤	-	11.20	-	11.48	-0.23	[-1.39,0.94]	-0.03
ASQ:SE-2e ¤	47.17	25.73	50.25	25.16	1.33	[-4.98,7.64]	0.04
Child height	56.99	70.39	57.09	70.42	0.01	[-1.31,1.34]	0.00
Child weight (kilo)	4.95	8.40	4.86	8.38	-0.01	[-.31,0.30]	-0.00
*Single Items*							
Loneliness	7.07	7.15	7.06	7.27	-0.15	[-1.04,0.75]	-0.03
Network	9.07	7.90	8.17	8.28	-1.07	**[-1.85,-0.28]**	-0.18
Confidants	9.61	9.74	9.06	9.51	0.19	[-0.20,0.57]	0.09
Overall health self-report	8.70	8.86	8.42	8.52	0.23	[-0.23,0.69]	0.09
Life satisfaction	9.16	9.13	9.06	8.95	0.15	[-0.20,0.50]	0.07
Budget	7.85	7.29	7.42	6.56	0.33	[-0.29,0.95]	0.07
Child temperament	8.87	9.25	8.56	9.29	-0.12	[-0.58,0.35]	-0.05
Child overall health	9.59	9.45	9.11	9.42	-0.04	[-0.46,0.38]	-0.02
Child enjoys reading	-	6.53	-	6.21	0.34	[-0.59,1.27]	0.07
Days reading	-	3.54	-	2.78	0.80	[-0.15,1.75]	0.15
Days singing	-	6.42	-	6.07	0.35	[-0.32,1.02]	0.11

**Bold** items are significant at *p* < 0.05; IYPB: Incredible Years Parents and Babies; UC: Usual Care; T1: Time 1 (pre-intervention); T2: Time 2 (post-intervention); β: regression estimate; CI: 95% Confidence Interval of regression estimate; *d*: Cohen’s d

¤: low score is favorable; KPCS: Karitane Parenting Confidence Scale; PSS: Parenting Stress Scale; MDI: Major Depression Inventory; WHO5: Well-Being Index; RSS: Rosenberg Self-Esteem Scale; MABISC: Mother and Baby Interaction Scale; ASQ:SE-2e: Ages and Stages Questionnaire: Social-Emotional-2 experimental version

### Differential Effects

We divided the sample into quarters and halves to look for moderating effects. We only performed the moderator analyses on the rating scales (KPCS, PSS, MDI, WHO5, RSS, MABISC, and ASQ:SE-2e), as the single items had very low variation and could not meaningfully be divided. Table 5 shows regression outputs for post-intervention outcomes for the following groups: mothers scoring within the lowest 25% and 50% at baseline, and mothers scoring within the highest 25% and 50% at baseline. For the lowest-performing 25%, parent confidence (KPCS) and mental health (WHO5) are significantly worse for the intervention group immediately following the intervention. For the lowest-performing 50%, parent stress (PSS) is significantly worse for the intervention group post-intervention. For both the lowest 50% and 25%, all other outcomes also trend in this direction, except for self-esteem (RSS) for the lowest 25% and depression (MDI) for the lowest 50%. In contrast, for mothers in the highest performing groups, all outcomes favor the IYPB group, except for parent confidence (KPCS) for the 25% highest, and depression MDI for the 25% and 50% highest; these results are not statistically significant apart from parent stress. For those who scored in the top 50% at baseline, parent stress (PSS) scores are significantly better for IYPB mothers than mothers who received UC

**Table 5:** Regression results of interaction analyses for mothers divided into groups based on pre-intervention (T1) scores of presented measures. Results compare mothers immediately following the intervention who received the IYPB program to those who received usual care within the groups scoring in the lowest 25^th^ and 50^th^ percentiles and the highest 50^th^ and 75^th^ percentiles.

	<25% at T1		<50% at T1		>50% at T1		>75% at T1	
	β	95% CI	β	95% CI	β	95% CI	β	95% CI
KPCS	-2.37	**[-4.45,-0.29]**	-0.49	[-2.09,1.12]	0.49	[-1.11,2.10]	-0.14	[-1.76,1.48]
PSS ¤ ±	8.21	[-0.10,16.51]	5.33	**[0.27,10.38]**	-6.11	**[-11.07,-1.14]**	-3.59	[-9.79,2.61]
MDI ¤	1.13	[-3.76,6.02]	-0.51	[-4.43,3.41]	0.49	[-3.43,4.41]	0.65	[-4.23,5.53]
WHO5	-18.62	**[-32.40,-4.84]**	-5.13	[-15.67,5.42]	4.75	[-5.74,15.24]	4.90	[-7.25,17.05]
RSS	0.12	[-3.63,3.87]	-0.81	[-3.84,2.20]	1.03	[-1.97,4.03]	2.31	[-0.86,5.48]
MABISC ¤ ±	2.66	[-0.17,5.49]	1.06	[-1.42,3.55]	-1.10	[-3.57,1.38]	-1.29	[-4.63,2.04]
ASQ:SE2e ¤	0.96	[-16.05,17.98]	9.26	[-3.19,21.71]	-9.26	[-21.71,3.19]	-4.98	[-17.71,7.74]

**Bold** items indicate significant interaction effect at *p* < 0.05; T1: Time 1; β: regression estimate for interaction term; CI: 95% Confidence Interval of regression estimate

¤: low score is favorable

±: KPCS score at baseline used for group

KPCS: Karitane Parenting Confidence Scale; PSS: Parenting Stress Scale; MDI: Major Depression Inventory; WHO5: Well-Being Index; RSS: Rosenberg Self-Esteem Scale; MABISC: Mother and Baby Interaction Scale; ASQ:SE-2e: Ages and Stages Questionnaire: Social-Emotional-2 experimental version

### Clinical Levels

Table 6 shows the proportion of mothers within IYPB and UC with clinical levels for outcomes with thresholds for clinical levels. There is no difference in the proportions between IYPB and UC for any of the outcomes. For all outcomes, the proportion of mothers with clinical levels falls between T1 and T2.

**Table 6:** Proportion of mothers who scored with in the clinical range at pre-intervention (T1) and post-intervention (T2) for those who participated in the IYPB program and in usual care (UC).

	T1		T2	
	% clinical		% clinical	
	IYPB	UC	IYPB	UC
KPCS	24	33	10	6
MDI	5	8	3	6
WHO5	4	11	1	0
RSS	4	3	3	3

KPCS: Karitane Parenting Confidence Scale; MDI: Major Depression Inventory; WHO5: Well-Being Index; RSS: Rosenberg Self-Esteem Scale

### Sensitivity analyses

Sensitivity analyses included OLS regression without imputation, random effects modeling, and difference-in-differences estimation on the imputed data, as presented in Table 7. The results presented here differ from those in Table 4 only in that, for the OLS regression without imputed data, mothers who participated in the IYPB group reported reading significantly more days per week to their infants than control mothers.

**Table 7:** Sensitivity analyses comparing OLS regression with and without imputation, ANCOVA, random effects modeling, and difference-in-differences estimation at post-intervention for IYPB and usual care (UC) mothers.

	**OLS-I**	**OLS**	**ANCOVA**	**RE**	**DiD**
	*N =* 110	*N* = 102	*N* = 204	*N* = 220	*N* = 220
KPCS	-0.08	-0.02	0.49	-0.48	-0.48
MDI	0.36	0.37	2.26	1.57	1.57
WHO	-1.48	-1.48	1.69	-4.45	-4.45
RSS	-0.17	-0.07	0.83	-0.75	-0.75
PSS	0.79	0.61	-	-	-
MABISC	-0.23	-0.33	-	-	-
ASQ:SE-2e	1.33	1.09	0.45	3.67	3.67
*Single items*					
Loneliness	-0.15	-0.10	0.00	-0.13	-0.13
Network	-1.07**	-0.95*	7.64**	-1.28**	-1.28**
Confidants	0.19	0.20	1.07	-0.32	-0.32
Overall health self-report	0.23	0.19	0.06	0.05	0.05
Life satisfaction	0.15	0.17	0.06	0.08	0.08
Economy	0.33	0.31	0.85	0.30	0.30
	*N* = 112	*N* = 104	*N* = 208	*N* = 224	*N* = 224
Child weight (kg)	-0.01	0.02	0.10	-0.07	-0.07
Child height (cm)	0.01	0.31	0.10	-0.00	-0.00
Child temperament	-0.12	-0.14	1.41	-0.36	-0.36
Child overall health	-0.04	-0.08	3.31	-0.44	-0.44
Child enjoys reading	0.34	0.54	-	-	-
Days reading	0.80	0.94*	-	-	-
Days singing	0.35	0.41	-	-	-

* *p* < 0.05

** *p* < 0.01

*** *p* < 0.0001

OLS-I: ordinary least squares regression with imputed data; OLS: ordinary least squares regression without imputed data; ANCOVA: analyses of covariance, RE: random effects, DiD: difference-in-differences

### Implementation and Treatment Fidelity

After each session, the group leaders completed the checklists provided. Although most items were applied during the sessions, sometimes elements were postponed due to time constraints. The group leaders usually included two to four vignettes in each session, but one session included four to six. As a result of parent feedback, the group leaders changed some elements of the program to fit the Danish context (i.e., some vignettes and exercises were dropped because parents felt that they were not relevant to their situations). The group leaders also spaced the sessions so that the infants were old enough for the last sessions to be age-relevant.

### Parent and Group Leader Satisfaction

An evaluation of user satisfaction with the program was completed for families who took part in the IYPB intervention in Ikast-Brande; not all families who were evaluated in the satisfaction study participated in the trial Almost half of the parents stated that attachment to their child was not influenced by participating in IYPB, while 36% stated that it was improved [69]. Only 1% reported low parenting confidence at the end of the intervention. The elements of the program that parents liked the most were group discussions, talking with others, and sharing experiences. The video vignettes were the least favoured element (25% felt that they were appropriate or very appropriate). Less than half of the parents (42%) felt that issues around babyproofing the home were appropriate or very appropriate. Overall, parents preferred the first sessions. Parents also reported that because their name appeared on the evaluation form, they were not always as critical as they would have been on an anonymous evaluation.

Common reasons for participating in the IYPB program were that parents had a general wish to be a good parent and that they were afraid that they might miss out on important information if they did not participate [70]. Parents’ expectations were not always fulfilled, as some had expected the sessions to involve more direct teaching and advice from the health visitors. Some parents statedthat the program structure was a bit too rigid, and they would have sometimes preferred to discuss other issues that they felt were important. Some parents stated that the information given was too basic for their level of knowledge, these reports may have come from mothers who had higher levels of functioning as the program was developed for lower functioning families and families of all levels participated in the groups. Some suggested that the sessions would have been satisfactory had they only involved the mothers, without group leaders. About half of the parents said they would recommend or strongly recommend the program to other parents.

The group leaders generally felt satisfied with the running of the IYPB groups, although some mentioned that they would have appreciated more acknowledgment from their leaders. Group leaders reported that group dynamics differed according to the specific participants. If parents shared insecurities or worries during the first few sessions, group leaders reported that their doing so opened up the dialogue for the remaining sessions. In groups where parents did not share such feelings, the group leaders reported that the more insecure parents stopped coming. Group leaders also reported that the group dynamics were also affected by the participation of fathers and that it was generally difficult to getfathers to participate.

### Cost

We were able to obtain cost data for only one site. Ikast Brande reports that the cost of IYPB per family is approximately DKK 7000 (≈$1045) when groups consist of eight families. The price includes training for group leaders, preparation time, housing, food and drink, supervision, and time for group sessions. No information was available on the cost of UC. As IYPB was offered in addition to UC, all mothers received UC. Compared to the cost analyses reported in the Wales evaluation [75], the price of running a group in Ikast-Brande is lower than the price for the initial Welsh group, but higher than that for subsequent Welsh groups.

## Discussion

In this pilot trial of the IYPB program as a universal intervention for parents with infants, there where no statistically significant differences on the measured outcomes between the IYPB and UC groups immediately following the intervention, apart from mothers’ social netvork. Mothers in the intervention group reported a smaller network to help them with practical issues than mothers in the control group. It seems unlikely that during the 10–20 week participation period in the IYPB program from pre to post intervention mothers’ available network would be diminished. An alternative explanation is that working to establish a support network for parents is a central focus of the IYPB program; as a result, intervention mothers may become more aware of the network that they currently have available to them and feel that they need to establish more support while control mothers may not have considered the importance of developing their network to the same degree. In addition, the mothers’ support network was a secondary outcome measured by a single item, and as all 20 outcomes at post-intervention were tested using a 5% significance level, the finding may be a spurious effect. This result should be re-examined at follow-up to determine if it is sustained.

Our findings are consistent with the results of the pre-post evaluation of IYPB in Wales, where Jones et al. did not find effects on parent report measures, but did find improved parent sensitivity in the IYPB group on parent-child observations. The baseline means of our primary outcome, parenting confidence (KPCS), and the change from T1 to T2 are nearly identical to those in the Jones et al. study [39]. Therefore, there is reason to believe that observed parent sensitivity may be improved in the present sample and those findings should be evaluated. The results are also similar to those of Evans et al. IYPB evaluation, which found statistically significant improvement from pre to post for parenting confidence (KPCS) and mental health. The KPCS baseline means in the Evans study are lower than in ours (38.66 compared to 41.09), and the change from T1 to T2 is larger (2.26-point improvement compared to 1.76), this is likely because the sample in the Evans study consisted of a disadvantaged group of mothers.

The lack of effect of IYPB as a universal intervention in the present pilot trial is consistent with some of the previously discussed disadvantages of universal interventions. For instance, it is more likely that the families with the greatest needs will decline to participate [76] and it is difficult to detect overall effects of universal interventions without very large populations due to the heterogeneity of the sample [77]. A recent systematic review of the effects of universal interventions for parents with infants on child development and the parent-child relationship found mixed results for the effect of universal interventions [78]. The systematic review includes three universal interventions discussed in the introduction: the Australian Toddlers Without Tears program [45,48], the Australian Face to Face program [49], and the US trial of parent training [50]. None of the three RCTs found intervention effects. For example, the authors of the Toddlers Without Tears program conclude, “A brief universal parenting programme in primary care is insufficient to prevent development of preschool externalising problems” [45]. Our results indicate that even the more intensive IYPB intervention, which starts when the infant is younger, has no effects immediately following the intervention for a universal population where the quality of usual care is high. It is important to note that the IYPB program was developed for disadvantaged families who showed observable difficulties in caring for their infants, not for a universal population.

There are several possibilities for the results of our findings. The sample is a universal group of mothers with a relatively high education level. These mothers would be expected to do well caring for their infants without the intervention, and their baseline scores were higher than those of disadvantaged groups.

A common issue for evaluations conducted with universal samples is the ceiling effect [79]. This effect applies to some of the outcomes in this trial, including one of the primary outcomes (KPCS). A marked ceiling effect means that measuring improvement over time is difficult. However, when looking at all mothers in the trial as a whole, we find that almost all outcomes (including the KPCS) significantly improve from T1 to T2. The significant negative effects that we find are related to measures that do not have marked ceiling or floor effects (PSS and WHO-5).

It is important to consider when interpreting the results that the control group received usual care in Danish health centers, which is a relatively extensive intervention. Almost all Danish families take advantage of five or six free home visits from a health visitor in addition to three free scheduled child-health visits to their general practitioner within the child’s first year of life [53,54]. For most outcomes, the sample as a whole significantly improved over time, suggesting that the universal intervention already offered to families in Denmark is beneficial or that the parents in this population adjust well to their new roles over time. From the results of this trial, we cannot conclude whether or not IYPB would be superior to no intervention and we do not know what the effects of the intervention will be for the infants or the parents longer term.

The results indicate that there may be differential effects of the IYPB intervention. Contrary to our hypothesis, we find that mothers with the lowest scores at baseline may experience negative effects from IYPB. When the sample is divided into two groups using pre-intervention scores (lowest 50%- and highest 50%)- most outcomes favor the IYPB group for the highest functioning halfof the sample, whereas the opposite applies to all of the outcomes from the lowest functioning half. In the moderator analyses we find interaction effects for the parent stress (PSS) measure; for the highest-functioning half of the mothers, we find that the IYPB group show significantly lower parent stress (PSS). However, for those in the lowest functioning half of the sample at baseline, the mothers who received IYPB scores significantly worse on parent stress (PSS)immediately following the intervention. In addition, for those in the lowest-scoring quarter, parent confidence (KPCS) and general well-being (WHO5) are significantly worse for IYPB mothers than for UC mothers at post treatment. These results are consistent whether or not we conduct the analyses using the imputed data.

Group interventions are based on the assumption that participants benefit from being in a safe environment where they feel supported by others who share their concerns and difficulties [80,81]. If a group is comprised of parents with widely varying experiences of becoming a parent, these differences may reduce the level of cohesion within the group.Previous research has shown that the lowest-functioning members of a group can experience negative effects of group participation if the other members of the group function at a much higher level [82]. In this case, the mothers who reported finding parenting more challenging before the IYPB program began, may have felt even more insecure and less competent in their abilities after meeting with parents who felt confident, self-efficacious, and less stressed in their parenting roles. If the differences in parents functioning reduced group cohesion and participants feelings of acceptance, the intervention may actually contribute to increasing inequality, a point highlighted in discussions of the disadvantages of implementing universal rather than targeted interventions [77].

Two separate studies indicate that the parent and group leader satisfaction with the program could have also influenced our results[69,70]. Only about half the parents reported at the end of the intervention that they would recommend it to other families Both the parents and group leaders indicated in an evaluation of the implementation that the program may need to be modified somewhat to meet the needs of a universal group of parents.

This is a pilot trial and the results should therefore be interpreted with prudence. In addition, it will be important to consider the results of the parent-child interactions (from observational data) and 18-month follow-up data when these analyses become available. The outcomes could differ from the present study for the parent-child relationship quality when rated by objective observers and current outcomes may change over time.

## Conclusion

This study is the first RCT of the IYPB program as a universal intervention for parents with infants. There was relatively little difficulty rrecruiting families to the effectiveness l evaluation, and the majority of mothers randomized to the IYPB group participated in the intervention, even though not all reportedfeeling satisfied with the program. Apart from one item, which may be a spurious effect (IYPB mothers reported having a smaller network than UC mothers post-intervention), we found no difference between the groups on any outcomes immediately following the intervention. There were differential effects hen the sample was divided into the lowest- and highest-scoring halves on baseline scores; we found significantly improved parental stress scores for the highest functioning mothers and significant negative effects on parental stress and mental health at post-intervention for the IYPB mothers who were in the lowest-functioning groups at baseline. All other outcomes appear to be trending in this direction. Given these results and the feedback from parents and group leaders, it is possible the IYPB program may be more effective when compared with high quality usual care if some cultural adaptations are made for specific populations and if it is used with more homogenous parent groups (i.e., level of parental function). The IYPB intervention was developed for use with groups of lower functioning families rather than a universal population; future research should investigate the effects of the program with groups of parents (both high and low functioning) who have similar mental health and parenting experiences.

## Supporting Information

S1 File Consort Checklist. (PDF)

S2 File Study Protocol. (PDF)

S3 File Trial Protocol. (PDF)

S4 File Danish Protocol with translation. (PDF)
