# Supplementary material for: The Incredible Years Parents and Babies Program: A Pilot Randomized Controlled Trial
Source: PLoS One. 2016 Dec 14;11(12):e0167592. doi: 10.1371/journal.pone.0167592 (PMC5156553; doi:10.1371/journal.pone.0167592)
Supplement: S3 File — (PDF) [file pone.0167592.s003.pdf]

FORSØGSPROTOKOL

Der udføres et randomiseret kontrolleret studie (RCT) af De Utrolige År babyprogram (DUÅ baby). Formålet med studiet er at undersøge effekten af indsatsen i en dansk kontekst.

DESIGN

Studiet udføres som et prospektivt, to armet parallelgruppe, assesorblindet, randomiseret kontrolleret forsøg, hvor deltagerne fordeles tilfældigt i to grupper – indsats- eller kontrolgruppe. Familier med børn, der falder inden for målgruppen til indsatsen, randomiseres til indsats- eller kontrolgruppe.

MÅLGRUPPE

Studiets målgruppe er forældre til babyer på 0-4 måneder. Barnet må ikke være anbragt uden for familien. Eksklusionskriterier er alvorligt handikap ved barnet, mental retardering ved forældre eller barn, alvorlig psykisk lidelse ved forældre eller massivt misbrug ved forældre.

DELTAGENDE KOMMUNER

Herning og Ikast-Brande kommuner indgår i studiet. I Herning rekrutteres der alle mødre i nogle distrikter af kommunen hvorimod Ikast-Brande rekrutterer førstegangsmødre. Der er udarbejdet informationsmateriale til både dem, der rekurtterer og generel information om studiet til kommunen.

REKRUTTERING AF FAMILIER

Rekruttering til indsatsen foregår igennem forvaltningen, enten via jordemoder, sundhedsplejerske eller sagsbehandler. Der udleveres en informationsfolder til familierne. Desuden kan en Youtube video, hvor studiet præsenteres, vises for familierne. Alle familier, der falder inden for målgruppen skal inviteres til at deltage i studiet. De deltagende familier, vil få 200 kr. pr. besvaret spørgeskema i form af et gavekort, som kompensation for at udfylde skemaet. Både mor og far inviteres til at udfylde spørgeskemaet. Far udfylder på familiens egen computer. Familierne vil blive forsøgt fastholdt i studiet ved at sende nyhedsmails, julekort og lignende.

SAMTYKKE

Deltagere skal informeres både skriftligt og mundtligt om studiet, og der indhentes skriftligt samtykke fra mor og eventuelt også fra far. Deltagere vil blive informeret om, at eventuel deltagelse i studiet ikke vil få indflydelse på deres fremtidige behandling, og at de til enhver tid kan trække sig ud af studiet. Studiet anmeldes til Datatilsynet og Den

Lokale Videnskabsetiske Komite. Studiet registreres ved clinicaktrials.gov inden første familie rekrutteres og forsøgsprotokollen publiceres inden sidste familie bliver randomiseret.

INTERVENTION OG KONTROL

Interventionen består af DUÅ baby, som er en manualbaseret behandling med en varighed på 8 uger jf. den mere detaljere beskrivelse af programmet i projektbeskrivelsen.

Kontrolgruppen vil fx få tilbudt den behandling man ellers vil give målgruppen (også kaldet treatment as usual, eller TAU). TAU omfatter det almindelige sundhedsplejerske tilbud og andre foranstaltninger så som ekstra sundhedsplejerske besøg eller familiebehandling hvis der er behov for det.

Det er muligt, at TAU vil variere imellem de to kommuner. Kommunen vil blive bedt om at registrere hvilken form for behandling familierne i TAU gruppen modtager og hvordan den forløber (fx hvor mange timer om ugen over hvor lang tid) for at kunne give et retvisende billede af hvad TAU behandlingen omfatter og hvordan den adskiller sig fra DUÅ baby behandlingen.

ETISKE OVERVEJELSER

Det vurderes at være etisk forsvarligt at fordele familierne i indsats- og kontrolgruppe, da man ikke på nuværende tidspunkt har undersøgt, om indsatsen har en positiv effekt for de deltagende familier. De familier, der kommer til at fungere som kontrolgruppe vil modtage enten en anden indsats eller den standardbehandling man ellers ville have givet dem før babyprogrammet blev taget i brug. Et af formålene med at lave effektmålingen af babyprogrammet er at undersøge, om indsatsen eventuelt kunne have en negativ effekt, altså gøre de deltagende familier dårligere. Dette er særligt vigtigt at undersøge, da det vil være meget problematisk, hvis familier bliver dårligere af at deltage i en socialpolitisk indsats designet til at hjælpe og støtte familien.

RANDOMISERING

Familier randomiseres til indsats- og kontrolgruppe via simpel blok randomisering i forholdet 2:1 (DUÅ Baby:TAU). Dette var et ønske fra kommunen da de gerne vil tildyde indsatsen til så mange som muligt. Hver kommune får deres egen blok (blokstørrelse 3) for at sikre lige mange i indsats og kontrol i hver kommune. Sagsbehandlere og behandlere ved ikke præcist hvordan randomiseringen foregår, da det kan give dem mulighed for at regne ud hvad næste familie vil blive tildelt. Randomiseringen foretages efter baselinemåling er udført og foretages af en uafhængig person. Randomiseringsroster med nummerering og allokering bliver kodet i Stata af uafhængig forsker og opbevares på sikkert drev.

Kontaktpersonen i kommunen sender via sikker forbindelse familiens kontaktinformation til forskeren som giver den videre til intervieweren. Intervieweren kontakter familien for at aftale tid til besøg 1. Intervieweren giver forskeren besked når interview er afholdt og familiens oplysninger gives derefter videre til sekretær/konsulent. Sekretær/konsulent randomiserer familien ved at indsætte familien på randomiseringsrosteren i den rækkefølge de kommer fra intervieweren. Kontaktpersonen i kommunen får herefter besked om hvad familien skal tilbydes.

BAGGRUNDSDATA

Baggrundsoplysninger om forældrene indsamles via spørgeskemaet og omfatter alder, uddannelse, arbejdsstatus, samlivsstatus, etnicitet, antal børn, alkohol- og stofmisbrug, rygning, fødselsvægt, længde og gestationsalder.

EFFEKTMÅL

Der måles ved baseline (T1), ved behandlingsafslutning (post-treatment T2) og ca. et år efter endt behandling når barnet er 18 måneder (follow-up T3) for at måle om en eventuel positiv effekt holder over tid (langtidseffekter).

Spørgeskemaundersøgelsen vil blive gennemført som hjemmebesøg af SFI Survey, der er kompetent til at foretage hjemmebesøg blandt alle befolkningsgrupper. Spørgsmålene vil blive udfyldt ved hjælp af en computer, som intervieweren medbringer. Forælderen vil blive bedt om selv at udfylde skemaet på computeren. Hvis der er brug for hjælp, vil intervieweren hjælpe. Det tilsigtes, at forælderen er alene i rummet sammen med intervieweren, når skemaet udfyldes.

Følgende effektmål indsamles:

		T1 Baseline	T2 Post test	T3 Follow up
**Parent measures**				
Karitane Parenting Confidence Scale	KPCS	√	√	
Parental Stress Scale	PSS		√	√
Major Depression Inventory	MDI10	√	√	√
World Health Organization Well-Being Index	WHO5	√	√	√
Rosenberg Self-Esteem Scale	RSS		√	
Sense of Coherence	SOC13	√		√
Background questions: Age, education, occupation, ethnicity, number of children, household status, housing situation, household economy, substance abuse		√	√	√
Single items on parent health, parent life satisfaction, support and network.		√	√	√
**Child measures**				
Ages and Stages Questionnaire - Social-Emotional	ASQ-SE	√	√	√
Strengths and Difficulties Questionnaire	SDQ			√
Single items on child health and child temperament		√	√	√
**Parent-child measures**				
Video (15 minutes)	EAS/CARE-Index		√	
Single items on interactions with child			√	√

Relevante effektmål kan tilføjes til T2 eller T3 hvis det ønskes.

Registerdata

Udover de standardiserede test vil det senere være muligt at indhente registerdata fx i forhold til skolekarakterer, kriminalitet mm for at vurdere langtidseffekterne af indsatsen.

Blinding

Deltagerne vil blive forsøgt blindet for formålet med undersøgelsen. Det vil sige at i stedet for at præsentere studiet som en undersøgelse af DUÅ baby (hvor programmet bliver præsenteret mere positivt end de behandlinger der indgår i TAU) bliver det præsenteret, som et studie af de forskellige indsatser over for målgruppen, fordi vi ikke ved hvad der virker bedst.

Målinger vil blive foretaget af interviewere, som er blindet for deltagerens gruppetilhørsforhold. Det kan dog ikke afvises, at informanten oplyser intervieweren, om hvilken gruppe familien tilhører.

Data leveres blindet til analyse. Det vil fremgå, om familien tilhører gruppe 1 eller 2, men det vil ikke fremgå hvilken gruppe, der er indsats, og hvilken der er kontrol.

STYRKEBEREGNING

Der er ikke udført nogen egentlig styrkeberegning, da der endnu ikke foreligger nogle resultater fra de igangværende pilotundersøgelser, og det heller ikke er fastlagt hvilket mål, der er primæroutcome, da det afhænger af delundersøgelse 2. De eksisterende studier af De Utrolige År viser dog pæne effekter – mange studier har moderat til store effekter, fx (Axberg, Hansson, & Broberg, 2007; Bywater et al., 2009; Gardner, Burton, & Klimes, 2006; Hutchings et al., 2007).

Med en styrke på 80 procent, et signifikansniveau på 5 procent og en mellemstor effekt (Cohen's *d*=0,50) skal der bruges 120 familier i studiet. Med en lille effekt (Cohen's *d*=0,20) skal der bruges omkring 800 familier.

ANALYSE

Analyse vil blive udført i overensstemmelse med CONSORT guidelines. Analyser udføres med Stata eller R. Deskriptive data beskrives med gennemsnit, median, range, standard afvigelse og procent. Missing data vil blive behandlet via multiple imputation modeller hvis nødvendigt. Sammenligninger udføres med T-test eller Q^2 test. Analyser vil blive udført som Intention To Treat (ITT) hvor alle, der er randomiseret inkluderes i analysen. Effektmål analyseres med multipel regression, med kontrol for baseline score og eventualt kontrol for andre relevante variable. Der vil blive anvendt to-sidet test med 5% signifikansniveau og 95% konfidensintervaller vil blive rapporteret. Standard fejl vil blive clustret om gruppen i indsatsgruppen. Der udføres også Complier Average Treatment Effect (CACE) analyse. For at undersøge effekt af deltagelse vil vi se på forældre der har deltaget mindst 3 gange ud af 8 og forældre der har deltaget minimum 75% (6 gange). Vi forventer at se en større effekt for de forældre, der har den dårligste funktionsevne ved T1. Derfor vil vi se nærmere på følgende grupper: forældre med kliniske niveauer ved T1, forældre med de dårligste 25% scores og forældre med de dårligste 50% scores.

OMKOSTNINGER

I det omfang det er muligt, indsamles data fra de deltagende kommuner om prisen for både DUÅ baby behandlingen og for de forskellige behandlinger, der indgår i TAU, således at der foreligger data til en beregning af omkostningerne ved DUÅ baby relativt til andre typer af behandling.

FLOWCHART

Nedenfor er illustreret, hvordan studiet forløber. Af de familier der er i målgruppen vil der være nogle, som ikke ønsker at deltage. De som ønsker deltagelse, vil udfylde samtykkeerklæring. Gennem randomisering vil deltagere blive opdelt i indsats- og kontrolgruppe. Der udføres en baseline-måling, en måling ved behandlingsafslutning (posttest) samt follow up et år efter. Det kan forventes, at nogle af de personer, som har udfyldt samtykkeerklæring, alligevel vil afbryde forløbet eller skifte behandling. Uanset hvad der sker for deltagerne efter randomisering, analyseres der på alle, som er blevet randomiseret (Intention To Treat (ITT)).

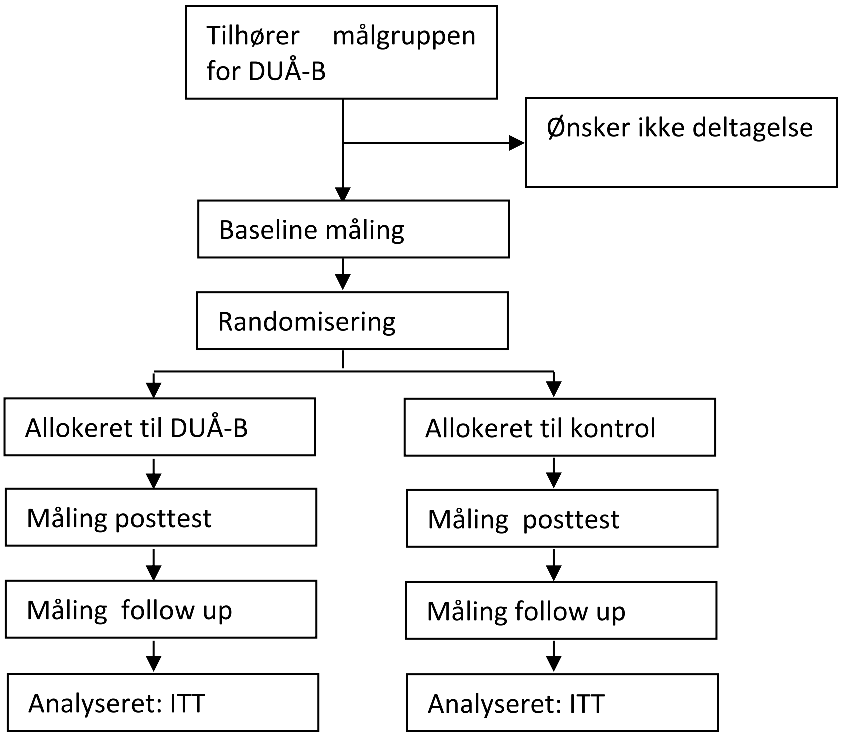

Axberg, U., Hansson, K., & Broberg, A. G. (2007). Evaluation of the Incredible Years Series - an open study of its effects when first introduced in Sweden. *Nordic Journal of Psychiatry*, *61*(2), 143–51.

Bywater, T., Hutchings, J., Daley, D., Whitaker, C., Yeo, S. T., Jones, K., … Edwards, R. T. (2009). Long-term effectiveness of a parenting intervention for children at risk of developing conduct disorder. *The British Journal of Psychiatry : The Journal of Mental Science*, *195*(4), 318–24.

Gardner, F., Burton, J., & Klimes, I. (2006). Randomised controlled trial of a parenting intervention in the voluntary sector for reducing child conduct problems: outcomes and mechanisms of change. *Journal of Child Psychology and Psychiatry*, *47*(11), 1123–1132.

Hutchings, J., Bywater, T., Daley, D., Gardner, F., Whitaker, C., Jones, K., … Edwards, R. T. (2007). Parenting intervention in Sure Start services for children at risk of developing conduct disorder: pragmatic randomised controlled trial. *BMJ*, *334*(7595), 678–678.
