# Supplementary material for: The Incredible Years Parents and Babies Program: A Pilot Randomized Controlled Trial
Source: PLoS One. 2016 Dec 14;11(12):e0167592. doi: 10.1371/journal.pone.0167592 (PMC5156553; doi:10.1371/journal.pone.0167592)
Supplement: S4 File — (PDF) [file pone.0167592.s004.pdf]

FORSØGSPROTOKOL

Der udføres et randomiseret kontrolleret studie (RCT) af De Utrolige År babyprogram (DUÅ baby). Formålet med studiet er at undersøge effekten af indsatsen i en dansk kontekst.

DESIGN

Studiet udføres som et prospektivt, to armet parallelgruppe, assesorblindet, randomiseret kontrolleret forsøg, hvor deltagerne fordeles tilfældigt i to grupper – indsats- eller kontrolgruppe. Familier med børn, der falder inden for målgruppen til indsatsen, randomiseres til indsats- eller kontrolgruppe.

UK: The study is a prospective, two-arm, parallel, assessoblinded, randomized controlled trial where participants are randomized to intervention or control. Families with children within the target group of the intervention are randomized to intervention or control.

MÅLGRUPPE

Studiets målgruppe er sårbare forældre til babyer på 3-5 måneder som vurderes egnede til at indgå i gruppeundervisning. Barnet må ikke være anbragt uden for familien. Eksklusionskriterier er alvorligt handikap ved barnet, mental retardering ved forældre eller barn, alvorlig psykisk lidelse ved forældre eller massivt misbrug ved forældre.

UK: The target group is vulnerable parents of babies 3-5 months old who can participate in a group intervention. The child must not be in out of home care. Exclusion criteria is serious physical or mental disability or massive drug abuse in parents.

Note: The trial was planned to recruit vulnerable families as stated here. But right at the time when recruitment started both local authorities changed their policies and decided to recruit all mothers – a universal sample. Therefore, the published protocol describes a universal group of parents and not a vulnerable sample.

REKRUTTERING AF KOMMUNER

For at studiet kan udføres skal de kommuner, der anvender DUÅ baby acceptere at indgå i studiet. Processen vil foregå ved at der sendes et brev til socialcheferne i de kommuner, der visiterer familier til DUÅ baby, hvori de tilbydes at deltage i studiet. Der udarbejdes en lettilgængelig informationsfolder om studiet som vedlægges. Hvis kommunen ønsker det, afholdes der et møde med socialcheferne, hvor planerne for studiet præsenteres. I de kommuner, der siger ja til deltagelse, vil der senere blive afholdt et møde med sagsbehandlere og behandlere, så også de bliver præsenteret for studiet.

Hvis det er muligt indgås der skriftlige aftaler på chefniveau med kommunerne, hvor disse forpligter sig til at henvise et vist antal familier til studiet. Hvis kommunerne henviser det ønskede antal udløser det en økonomisk kompensation på 2.000 kr. pr. familie. Modellen med en skriftlig forpligtende aftale med kommunerne, der udløser en økonomisk kompensation ved opfyldelse, anvendes tilsyneladende med succes i et multicenter studie, der aktuelt udføres i Sverige.

REKRUTTERING AF FAMILIER

Rekruttering til indsatsen foregår igennem forvaltningen, enten via jordemoder, sundhedsplejerske eller sagsbehandler. Der vil blive udarbejdet en informationsfolder til familierne samt en Youtube video hvor studiet præsenteres, som kan vises for familierne. Derudover kan det overvejes, om forskeren skal ringe til familierne og informere om studiet. Alle familier, der falder inden for målgruppen skal inviteres til at deltage i studiet. De deltagende familier, vil få 200 kr. pr. besvaret spørgeskema i form af fx gavekort, som kompensation for den tid, de har brugt på at udfylde skemaet. Dette er gængs procedure i lignende studier og øger sandsynligheden for at familier udfylder de opfølgende skemaer, hvilket er afgørende for studiets gennemførsel. Familierne vil blive forsøgt fastholdt i studiet ved at sende nyhedsmails, julekort og lignende. I forhold til de rekrutterende medarbejdere (jordemødre, sundhedsplejersker og sagsbehandlere) kan der laves en konkurrence, hvor man deltager i en konkurrence om fx en Ipad med et lod pr. familie man rekrutterer. Dette skulle gerne øge sandsynligheden for at de henvender sig til familier i målgruppen.

UK: Families are recruited by health visitors, midwifes or caseworkers. Families receive an information leaflet and a youtube video with information on the study. All families who are within the target group are asked to participate. Families receive a 200 DKK gift card for each assessment.

SAMTYKKE

Deltagere skal informeres både skriftligt og mundtligt om studiet, og der indhentes skriftligt samtykke. Deltagere vil blive informeret om, at eventuel deltagelse i studiet ikke vil få indflydelse på deres fremtidige behandling, og at de til enhver tid kan trække sig ud af studiet. Studiet anmeldes til Datatilsynet og Den Lokale Videnskabsetiske Komite. Forsøgsprotokollen registreres eller publiceres inden første familie bliver randomiseret, fx ved www.clinicaltrials.gov

UK: Participants receive oral and written information on the study and give written consent to participate. Participants can withdraw at any time. The study will be registered at Datatilsynet (the Dansih data agency) and the local ethics committee. The trial is registered with clinicaltrials.gov and the protocol is approved before the first family is recruited.

Note: By Danish law a trial like this one with no medical intervention or any biological assessment does not require ethical approvement by the local ethics committee. Approval was asked for by the local ethics committee but we received a letter where they stated that the trial by Danish law did not need this approval. Ethics approval was granted by the Internal Review Board at the Danish National Centre for Social Research.

INTERVENTION OG KONTROL

Interventionen består af DUÅ baby, som er en manualbaseret behandling med en varighed på 10 uger jf. den mere detaljere beskrivelse af programmet i projektbeskrivelsen.

Kontrolgruppen vil fx få tilbudt den behandling man ellers vil give målgruppen (også kaldet treatment as usual, eller TAU). TAU kan fx omfatte andre foranstaltninger inden for Servicelovens §52 som konsulentbistand, anden form for familiebehandling, et aflastningsophold eller støtte kontaktperson.

Det forventes, at behandlingen i kontrolgruppen vil variere en del fra kommune til kommune. Der vil derfor blive udført uddybende interviews med de deltagende kommuner for nærmere at kunne beskrive, hvad TAU indeholder. Sagsbehandlerne vil blive bedt om at registrere hvilken form for behandling familierne i TAU gruppen modtager og hvordan den forløber (fx hvor mange timer om ugen over hvor lang tid) for at kunne give et retvisende billede af hvad TAU behandlingen omfatter og hvordan den adskiller sig fra DUÅ baby behandlingen.

UK: The intervention is 10 sessions of the Incredible years parents and babies program. Control group will receive care as usual which is extected to vary between local authorities. Local authorities are asked to register what is offered as UC.

Note: The published protocol says 8 sessions as this was what was offered

ETISKE OVERVEJELSER

Det vurderes at være etisk forsvarligt at fordele familierne i indsats- og kontrolgruppe, da man ikke på nuværende tidspunkt har undersøgt, om indsatsen har en positiv effekt for de deltagende familier. De familier, der kommer til at fungere som kontrolgruppe vil modtage enten en anden indsats eller den standardbehandling man ellers ville have givet dem før babyprogrammet blev taget i brug. Et af formålene med at lave effektmålingen af babyprogrammet er at undersøge, om indsatsen eventuelt kunne have en negativ effekt, altså gøre de deltagende familier dårligere. Dette er særligt vigtigt at undersøge, da det vil være meget problematisk, hvis familier bliver dårligere af at deltage i en socialpolitisk indsats designet til at hjælpe og støtte familien.

RANDOMISERING

Familier randomiseres til indsats- og kontrolgruppe via simpel blok randomisering. Hver kommune får deres egen blok for at sikre lige mange i indsats og kontrol i hver kommune. Der vil være variabel blokstørrelse. Sagsbehandlere og behandlere må ikke vide præcist hvordan randomiseringen foregår, da det giver dem bedre mulighed for at regne ud hvad næste familie vil blive tildelt. De kan fx få fortalt at SFI via computer sørger for at det er tilfældigt, hvilken behandling familien bliver tildelt. Randomiseringen foretages efter baselinemåling er udført og foretages af en uafhængig person.

I udgangspunktet randomiseres der 1:1 så indsats- og kontrolgruppe bliver lige store. Der er dog overvejelser om at randomisere 2:1 for at imødekomme kommunerne, da de ofte ønsker at så mange som muligt skal tilbydes interventionen. Man vil miste en smule styrke ved at gøre dette, men det vil give bedre chancer for at rekruttere.

UK: Families are randomized by simple randomization, stratifiying for site and with blocking. Practitioners must not know the exact randomization rules. They will be informed of the allocation by SFI. Randomization will take place after baseline assessment and will be performed by an independent person. We expect to use a 1:1 randomization but may use a 2:1 (IY:UC) randomization as the local authorities wish to offer the intervention to as many families as possible. There will be a minor loss in power from doing this.

Note: We decided to use the 2:1 allocation to make sure that local authorities had enough families to start a group.

Effektmål – standardiserede test

Effektmål vil blive udvalgt så de stemmer overens med hovedformålet med indsatsen og vil så vidt muligt blive udført med standardiserede test, der har vist gode måleegenskaber. Dette gør det blandt andet muligt at sammenligne effekten med eventuelle andre studier. Der måles ved baseline (førmåling), ved behandlingsafslutning (posttest) og ca. et år efter endt behandling når barnet er fyldt 1½ år (follow up) for at måle om en eventuel positiv effekt holder over tid (langtidseffekter). Der måles når barnet er fyldt 1½ år, fordi det først er ved den alder, at man kan bruge de meget anvendte test til adfærd som fx Child Behavior Checklist (CBCL).

UK: Outcomes will be measured at post intervention and when the child is around 1½ years old.

Barnets udvikling vil blive undersøgt fx ved hjælp af vækst kurver (vægt og højde fra lægeundersøgelser/sundhedsplejerskebesøg). Der kan eventuelt anvendes en standardiseret test som Ages and Stages Questionnaire (fra 6 måneder) til at vurdere barnets udvikling.

Relation og tilknytning mellem mor og barn vil blive målt.

Moderens velbefindende vil blive målt i forhold til generel trivsel (fx WHO5), niveau af depression og generel psykisk velbefindende (fx Symptom Checklist SCL25). Familiens stressniveau, forældrenes alliance og forældrenes tro på egne evner vil også blive målt, fx med Tool to Measure Parenting Self-Efficacy (TOPSE).

Spørgeskemaundersøgelsen vil blive gennemført som hjemmebesøg af SFI Survey[1] eller en lignende organisation, der er kompetent til at foretage denne type dataindsamling blandt udsatte befolkningsgrupper. Spørgsmålene vil blive udfyldt ved hjælp af en computer, som intervieweren medbringer. Forælderen vil blive bedt om selv at udfylde skemaet på computeren. Hvis der er brug for hjælp, vil intervieweren hjælpe. Det tilsigtes, at forælderen er alene i rummet sammen med intervieweren, når skemaet udfyldes.

UK: Data will be collected through home visits. We will measure child development, parent-child relationship, parent mental health, parent stress, parent competence.

Registerdata

Udover de standardiserede test vil der også blive anvendt registerdata i det omfang det er muligt forhold til at se om der her kan ses en effekt af indsatsen. Dette kan fx omfatte fødselsvægt samt udvikling i forhold til vægt og højde, forældre og barns forbrug af sundhedstilbud, andre sociale foranstaltninger, anbringelse uden for hjemmet. Man vil senere kunne indhente registerdata fx i forhold til alder ved skolestart, test i skole, kriminalitet mm for at vurdere langtidseffekterne af indsatsen.

Baggrundsdata

Baggrundsoplysninger om forældrene indsamles via spørgeskemaet og via data fra Danmarks Statistik. Disse data omfatter fx alder, indkomst, uddannelse, socioøkonomisk stilling, samlivsstatus, psykiske og somatiske lidelser, domme for kriminalitet og misbrugsbehandling (oplysninger om misbrug indhentes eventuelt fra Sundhedsstyrelsens registre, som er hurtigere opdateret).

Blinding

Deltagerne vil blive forsøgt blindet for formålet med undersøgelsen. Det vil sige at i stedet for at præsentere studiet som en undersøgelse af DUÅ baby (hvor programmet bliver præsenteret mere positivt end de behandlinger der indgår i TAU) bliver det præsenteret, som et studie af de forskellige indsatser over for målgruppen, fordi vi ikke ved hvad der virker bedst.

[1]. SFI Survey er en selvstændig del af SFI, der drives på kommercielle vilkår. SFI Survey er meget erfarne i at levere data til forsknings- og analyseformål og har et landsdækkende interviewerkorps med 350 interviewere.

Målinger vil blive foretaget af interviewere, som er blindet for deltagerens gruppetilhørsforhold. Det kan dog ikke afvises, at informanten oplyser intervieweren, om hvilken gruppe familien tilhører.

Data leveres blindet til analyse. Det vil fremgå, om familien tilhører gruppe 1 eller 2, men det vil ikke fremgå hvilken gruppe, der er indsats, og hvilken der er kontrol.

UK: Assessors are blinded – but families may reveal their intervention status at home visits. Data analyst is blinded.

Styrkeberegning

Der er ikke udført nogen egentlig styrkeberegning, da der endnu ikke foreligger nogle resultater fra de igangværende pilotundersøgelser, og det heller ikke er fastlagt hvilket mål, der er primæroutcome, da det afhænger af delundersøgelse 2. De eksisterende studier af De Utrolige År viser dog pæne effekter – mange studier har moderat til store effekter, fx (Fergusson, Stanley & Horwood, 2009; Gardner, Burton & Klimes, 2006; Hutchings m.fl., 2007; Scott m.fl., 2010).

Med en styrke på 80 procent, et signifikansniveau på 5 procent og en mellemstor effekt (Cohens d=0,50) skal der bruges 120 familier i studiet. Dette er derfor udgangspunktet for studiet. Der vil blive udført en egentlig styrkeberegning på et senere tidspunkt, når der foreligger flere informationer.

UK: Samplesize: There are no data available on the intervention to inform samplesize calculations. Existing studies of the IY shows moderate to large effects. With power=80%, 5% significancelevel, and a medium effectsize (Cohens d=0,50) 120 families are needed.

Note: This sample size calculation was based on a vulnerable population – as the population for the trial was changed right at the time when recruitment started we had to redo the calculations. This led to a change in the trial status to a pilot trial as sample size could not be extended from the 120 that the funding covered.

Analyse

Analyse vil blive udført ved variansanalyse eller regression. Der vil blive kontrolleret for relevante baselineoplysninger. Analyser vil blive udført som Intention To Treat (ITT) hvor alle, der er randomiseret inkluderes i analysen. Missing data vil blive behandlet via fx multiple imputation modeller (Sainani, 2010). Der udføres også Complier Average Treatment Effect (CACE) analyse(Cook & DeMets, 2008).

UK: Analyses will be analyses of variance or regression analyses. We will control for relevant baseline measures. All analyses will be ITT. Missing data will be handled with multiple imputation. We will also perform CACE analyses.

PILOTSTUDIE

Forud for effektstudiet foretages et pilotstudie. Formålet med dette studie er at finde frem til om procedurer og test fungerer og om der er praktiske hindringer i forsknings-designet. I pilotstudiet undersøges rekrutteringsprocessen og der gennemføres et min-dre antal målinger med samme instrumenter og effektmål, som i det planlagte effekt-studie. Pilotstudiet kan også give en ide om de antagelser som ligger til grund for en eventuel styrkeberegning er korrekte. Efter pilottesten tilrettes studiet, hvis der er be-hov for det.

OMKOSTNINGER

I det omfang det er muligt, indsamles data fra de deltagende kommuner om prisen for både DUÅ baby behandlingen og for de forskellige behandlinger, der indgår i TAU, såle-des at der foreligger data til en beregning af omkostningerne ved DUÅ baby relativt til andre typer af behandling.

FLOWCHART

Nedenfor er illustreret, hvordan studiet forløber. Af de familier der er i målgruppen vil der være nogle, som ikke ønsker at deltage. De som ønsker deltagelse, vil udfylde sam-tykkeerklæring. Gennem randomisering vil deltagere blive opdelt i indsats- og kontrol-gruppe. Der udføres en baseline-måling, en måling ved behandlingsafslutning (posttest) samt follow up et år efter. Det kan forventes, at nogle af de personer, som har udfyldt samtykkeerklæring, alligevel vil afbryde forløbet eller skifte behandling. Uanset hvad der sker for deltagerne efter randomisering, analyseres der på alle, som er blevet randomi-seret (Intention To Treat (ITT)).

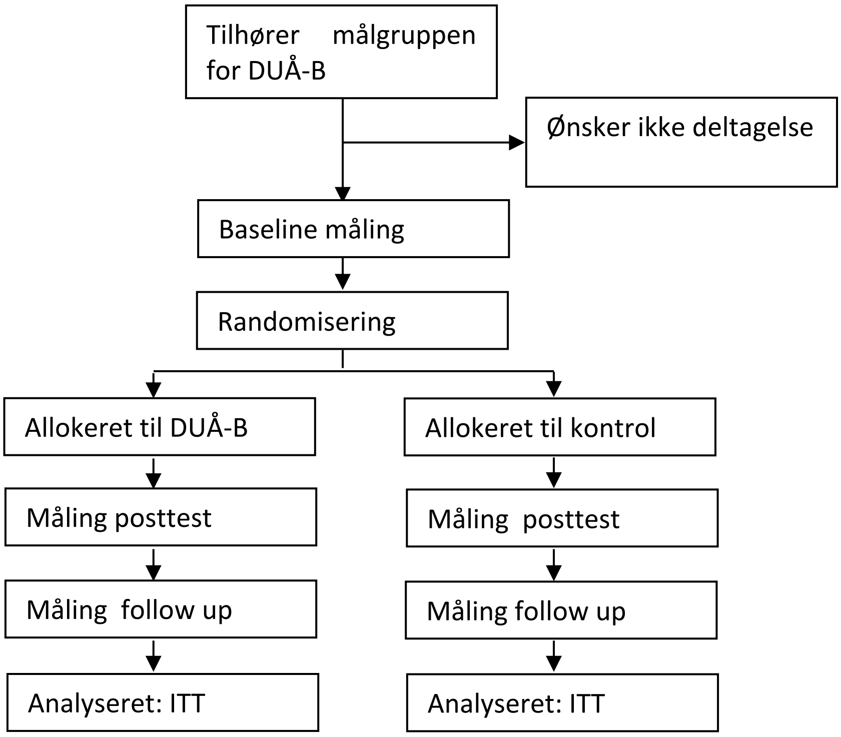

REFERENCER

Cook, T.D. & D.L. DeMets (2008): *Introduction to statistical methods for clinical trials.* CRC Pr I Llc.

Sainani, K.L. (2010): "Making sense of intention-to-treat". *PM.R*, *2*(3), s. 209-213.
